# Acceptability and feasibility of a school-based contraceptive clinic in a low-income community in South Africa

**DOI:** 10.1017/S1463423618000762

**Published:** 2019-03-20

**Authors:** Nondumiso Khoza, Phindile Zulu, Maylene Shung-King

**Affiliations:** 1 Ukwanda Center for Rural Health, Faculty of Medicine and Health Sciences, Stellenbosch University, Cape Town, South Africa; 2 Faculty of Medicine and Health Sciences, Stellenbosch University, Cape Town, South Africa; 3 University of Cape Town, Cape Town, South Africa

**Keywords:** clinic, contraceptives, schools, South Africa, teenage pregnancy

## Abstract

**Aim:**

To investigate how acceptable and feasible a school-based contraceptive clinic (SBCC) would be in a low-income South African community.

**Background:**

Teenage pregnancy is an important issue in South Africa, with significant health and social consequences. Issues regarding lack of confidentiality in an intimate community, unwelcoming health workers, long distances to clinics and perceptions of contraceptive side effects may all inhibit contraceptive use by adolescents. Although SBCC has been initiated and investigated in other countries, this approach is inadequately researched in South Africa.

**Methods:**

A mixed method study was conducted to assess the attitudes of one community towards establishment of an SBCC in their area. Methods of data collection included: focus group discussions (FGDs) with teenage girls from a local high school; a key informant interview with the school principal; a structured survey, including open-ended questionnaires with randomly selected parents of teenage girls from the same community; and a documentary analysis to explore relevant legal and policy considerations.

**Findings:**

Teenage girls, the school principal and parents with teenage daughters largely supported the idea of an SBCC, but with concerns about confidentiality, the possibility of increased promiscuity and contraceptive side effects. While legal statutes and policies in South Africa do not pose any barriers to the establishment of an SBCC, some logistical barriers remain.

## Background

Teenage pregnancy rates in South Africa, referring to girls aged between 13 and 19 years, remains high. The most recent South African Demographic and Health survey released in 2017, whilst reporting little difference in teenage pregnancy rates between the 1998 and the 2016 surveys, revealed that approximately 16% of girls between the ages of 15 and 19 had begun childbearing and at the time of the survey, 12% had given birth to a live infant (Statistics South Africa, 2016). In a two-year follow-up of 819 women aged 15–18 in South Africa’s Eastern Cape Province, 174 pregnancies were reported of which only 3.6% were wanted (Christofides *et al*., 2014). The South African rate in 2014 of 47 pregnancies per 1000 girls aged 15–19, was considerably higher when compared to neighbouring countries such as Botswana at 31 and other low- and middle-income countries like China, at 7, or Morocco at 24, but faring better than Brazil and Mexico at 67 and 62, respectively (The World Bank, 2014), suggesting a complex interplay of factors beyond country-level socio-economic parameters. Alcohol use by teenage girls, lack of access to contraceptives, gender inequalities and living in poverty are among the reported drivers of teenage pregnancy (Willan, 2013; Christofides *et al*., 2014).

Early childbearing is associated with far-reaching health and social consequences for teenage mothers and their babies, making pregnancy and childbirth complications the leading causes of death for 15- to 19-year-old girls (World Health Organization, 2014). Teenage mothers are at higher risks for eclampsia, puerperal endometritis and systemic infections (Ganchimeg *et al*., 2014). Associated perinatal complications include preterm and low-birth-weight babies. The stillbirth rate in adolescents is 5.1% compared to 0.9% of adult females (Chaudhuri *et al*., 2012). Teenage mothers also face greater socio-economic disadvantages and have fewer years of schooling, further deepening their poverty level. Children born to teenage mothers are often exposed to neglect and abuse and are more likely to end up being teenage mothers themselves, thus perpetuating the cycle of health and social risks (Brodmann and Douglas, 2016).

Despite the significant risks of teenage pregnancy, the prevalence of contraceptive use amongst sexually active adolescent women (15–24 years) in South Africa is reported at only 64.2% (Statistics South Africa, 2016). This is compounded by the reported reluctance of male partners to use condoms (Abdool Karim *et al*., 2014), thus leaving the girls with the sole responsibility for ensuring protection against pregnancy and sexually transmitted infections. Contributing to the low contraceptive usage are the many barriers to accessing contraceptive services for teenagers, in particular for those who are still at school. Clinic operating times do not favour schooling girls, and those who do make it to the clinics after school, reportedly experience negative attitudes from nursing staff (London *et al*., 2013).

In an attempt to strengthen struggling school health services, the Integrated School Health Policy (ISHP) was released in South Africa in 2012 (Department of health and Department of Basic Education, 2012). Amongst the proposed interventions, the ISHP requires the provision of sexual and reproductive health (SRH) services at schools by school health nurses, as part of an on-site service package. The services are intended to perform dual protection from both pregnancy and sexually transmitted infections. The ISHP states that learners who are older than 14 years may access contraceptives; but that they should be advised to inform and discuss their treatment with their parent or caregiver. The ISHP requires school health nurses to provide information on contraceptive methods; distribute condoms and refer students to the nearest clinic for additional contraceptive and other sexual health services (Department of Health, 2012).

School-based health services exist in a number of high- and middle-income countries, but vary in scope and function. Countries such as Hong Kong, Japan and the United States, have a one-nurse-one-school service, which provides a range of services, including SRH care (Lee, 2011). The United States appears to have the most consistent school-based SRH service model, but also with wide interstate variation (Brindis *et al*., 2012). In general, low- and middle-income countries have limited access to school-based health services, given their resource constraints.

School-based SRH clinics have reportedly reduced access barriers and hospitalization of teenage girls, as well as lowered transport costs to access local clinics (Crisp *et al*., 2012). The availability of school-based contraceptives has also reportedly promoted earlier commencement and more consistent use of hormonal contraceptives (Daniels *et al*., 2001). However, there is conflicting evidence about whether the rate of teenage pregnancy is reduced by school-based clinics (Davis *et al.*, 2012). Despite reported benefits, there has been some opposition to school-based clinics in the United States. Concerns include fears of promoting promiscuity and risking lack of confidentiality. Parents, further feel that providing contraception without their consent or knowledge would decrease involvement in their child’s health care. In contrast, an estimated 63% of parents endorse the prescribing and dispensing of contraceptives at school in one study (Alexander *et al*., 1992), while another dispelled any association between school-based contraceptive services and increased sexual activity among learners (Ethier *et al*., 2011).

In South Africa, the distribution of condoms at public schools was met with resistance by the general public and this did not change with the release of the ISHP (Han and Bennish, 2009). The Department of Basic Education (DBE), despite being a co-signatory of the policy, subsequently declared that the provision of SRH and the distribution of condoms at school specifically, would be subject to the approval of each individual school governing body, thus leaving the availability of this service to the discretion and potential biases of school governing bodies (Gray and Vadwa, 2014). Whilst a subsequent DBE draft policy on HIV, STIs and TB firmly proposes the provision of SRH services in schools, the policy retains the provision of these being subject to school-governing body approval (DBE, 2015). As a result, the full spectrum of sexual and reproductive is primarily provided by primary health care clinics, which expose teenagers to the barriers alluded to earlier. The perspectives and concerns of South African parents and teenagers are yet to be fully explored.

Against this background, we explored the perspectives of teenage girls and parents with teenage daughters, on the acceptability and feasibility of a Secondary school-based contraceptive clinic (SBCC). This small, exploratory, study was conducted in a low-income community outside the town of Worcester, which is located in a rural district in one of South Africa’s nine provinces. The community was purposely selected for a few reasons: the high teenage pregnancy rate (94.6 per 1000) (Western Cape government, 2012); the rural location of the community as very few studies on this subject are done in a rural setting and its proximity to the rural community placement site where the first author had spent a period of her rural placement during her final year of medical training. These converging factors provided a fortuitous opportunity to conduct this study. The focus is predominantly on the views of girls and parents with teenage daughters, as well as the policies and legal frameworks that govern contraceptive dispensing in South Africa.

**Figure 1:**
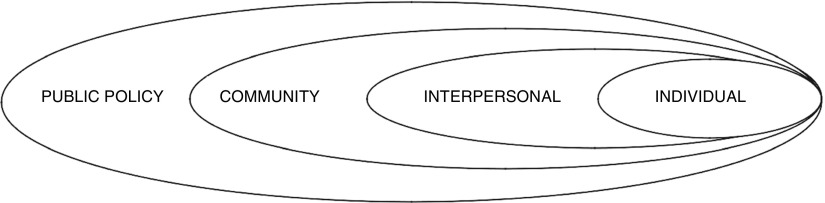
Social–ecological framework

We drew on the Social Ecological model (Figure 1) (Earp and Golden, 2012) as a guiding framework, given the complex and multifaceted factors alluded to earlier when considering teenage sexual behaviour. This theory posits that no single factor is responsible for human behaviour. It highlights the importance of exploring multifaceted and interactive effects of personal and environmental factors that influence behaviours and to consider these when developing organizational leverage points and intermediaries to facilitate health promotion.

## Methods

There are four components to the Social Ecological framework that potentially influence the acceptance and success of an SBCC: individual, interpersonal, community and public policy. The factors explored in this study were individual perspectives of teenage girls on teenage sexuality, pregnancy and the role of an SBCC, and perspectives from the broader parent community on these issues. Current public policy on school health and in particular the SRH component of school health services was examined through a documentary review.

We chose to focus on girls specifically, as they, unlike boys, are more likely to have required the full spectrum of SRH services for contraception requirements and pregnancy-related services. For our primary data collection, we employed mixed methods, comprising both qualitative and quantitative components. The qualitative component consisted of two focus group discussions (FGDs) with teenage girls in a conveniently selected secondary (high) school, which had a reportedly high teenage pregnancy rate. A key informant interview was held with the principal of the school in his capacity as the school leader and a de facto member of the school’s governing body. The quantitative part consisted of a structured questionnaire, administered to parents with teenage daughters. The documentary review was conducted on relevant South African government laws, policies and guidelines, primarily in the Departments of Health and Education.

We explored the perceptions of girls in the final Further Education and Training phase that spans Grades 10–12. Girls aged 16–19 were included in the study. The Department of Education divides schooling into four phases: the foundation phase of Grades 1–3; the intermediate phase of Grades 4–6; the senior phase of Grades 7–9 and the Further Education and Training phase of Grades 10–12). Girls in the study sample were in their final phase of schooling. Whilst South African law requires active parental consent for all research conducted with children 18 years and younger, current legal provisions allow for obtaining consent from adolescents to participate in research without parental consent, especially where research is assessed to have minimal risk. We opted to confine the research to the older age group, as older teenagers would feel more confident in participating without parental consent. As the study deals with perspectives on SRH; it may have introduced reluctance to participate if parental consent was required (Zuch *et al*., 2012). As these legal and ethical issues in South Africa regarding age of consent and in particular on matters sexual and reproductive are still subject to contestation and do not have absolute legal clarity, the researchers preferred to minimize any legal or ethical breaches (Strode *et al*., 2011; Zuch *et al*., 2012; South African Marketing Research Association, 2015).

Two FGDs lasting approximately 1 hour each were conducted using a topic guide wherein the questions were based on a theoretical framework of barriers and facilitators to the use of an SBCC as described by Attride-Stirling (Attride-Stirling, 2001).

The Life Orientation teacher at the school was asked to select a group of 18 participants aged 16–19 years; nine in each focus group. In an attempt to get a more representative sample, the teacher was asked to purposely select girls from varying socio-economic backgrounds and with varying levels of academic achievement. The participants were encouraged to speak in their home language and participation was voluntary. Written consent was obtained from all participants. FGDs were held in a private space on the school grounds, during the Life Orientation period. Discussions were recorded using a portable digital recorder and were transcribed, with translation into English where necessary by an independent translator. The topic guide included the following questions:

- Why do teenagers fall pregnant?
- How do you feel about contraceptives?
- Are there reasons why sexually active teenagers do not use contraceptives?
- What factors would encourage you to start using contraceptives?
- Do you believe teenagers have any problems when it comes to accessing contraceptives?
- How would you feel about an SBCC?
- What disadvantages do you think would be associated with an SBCC?
- What advantages do you think would be associated with an SBCC?

Thematic analysis was used to analyse results from the FGDs. Transcripts from FGDs were independently read by author NK and research supervisor MSK who then met and produced a deductive coding framework, resolving discrepancies by discussion and reaching consensus on the themes. Transcripts were then entered into Atlas-ti and coded. Text segments relevant to each theme were extracted, read as a whole and interpreted by NK and MV, using discussion to reach consensus.

A paper-based survey, which had a combination of structured and open-ended questions, sought to elicit views from community members who were parents to teenage daughters aged 16–19 years. Parents were not related to participants of the FGDs. We deliberately did not want to interview parents specifically related to the girls in the study, as the teenagers may have been reluctant to air their opinions if they knew their own parents were also linked to the study. Parents were selected by convenience sampling, from patients attending the clinic for chronic medication and from households close to the clinic. As insufficient numbers of parents at the clinic had teenage children aged 16–19 years, we sampled additional parents for interviews from surrounding households. Questionnaires were interviewer-administered unless the parents were not at home. When questionnaires were left behind; participants were asked to self-administer these and return it to the clinic. Assessments explored whether parents viewed teenage pregnancy as a problem in the area and how they felt about an SBCC being established in local secondary schools. Parents were asked to list possible advantages and disadvantages of an SBCC. Thematic analysis was done independently by two researchers (NK and PZ on responses to open-ended questions) and divergent coding and analyses were resolved though inter-researcher discussions. Simple frequencies were generated on responses for and against an SBCC, using an excel spreadsheet.

Lastly, analysis of legal and policy documents was done by the principal researcher (NK), using thematic analysis and guided by a data extraction sheet.

Ethics approval for the study was granted by the Human Research Ethics Committee of Stellenbosch University.

## Results

A total of 18 girls aged 16–19 years (nine in each group) participated in two FGDs, where they shared their opinions about contraceptives and the concept of an SBCC.

A total of 40 out of 45 parents with teenage daughters responded to the questionnaire-based survey. Parent’s ages ranged from 35 to 51 years and all respondents, except one, were female. Five parents were not at home and did not return the questionnaires to the clinic.

### Legal and policy imperatives for an SBCC

#### Legal provisions for an SBCC

One of the factors listed in the Social–Ecological Model that influences health, and healthcare is public policy. When considering an SBCC in South Africa, two pertinent Acts play a vital role in determining the obligation of health workers in relation to sexual activity amongst teenagers and the related access to contraceptives through an SBCC: The Children’s Act and the Criminal Law (Sexual Offenses and Related Matters) Amendment Act.

Section 134 of the Children’s Act states that contraceptives may be provided to the child on request from the child and without the consent of the parent or caregiver of the child if:

- ‘the child is at least 12 years of age;
- proper medical advice is given to the child; and
- a medical examination is carried out on the child to determine whether there are any medical reasons why a specific contraceptive should not be provided to the child’. (Children’s Act 38 of 2005)

This act allows for service providers to legally dispense contraceptives to school girls above the age of 12, without parental consent, thus posing no barrier to the establishment of an SBCC.

On the other hand, the Criminal Law (Sexual Offences and Related Matters) Amendment Act 32 of 2007 criminalizes the performance of certain consensual sexual acts (by adults and children) with children who are between 12 and 16 years of age. Section 15 of the Act imposes criminal liability for committing ‘statutory rape’:

‘Acts of consensual sexual penetration with certain children (statutory rape) 15 (1) A person (“A”) who commits an act of sexual penetration with a child

(“B”) is, despite the consent of B to the commission of such an act, guilty of the offence of having committed an act of consensual sexual penetration with a child’.

(Criminal law (Sexual Offenses and Related Matters) Act 32 of 2007: 13)

Section 54 of the Sexual Offences Act creates an obligation to report sexual offences against children and failure to do so could result in imprisonment:

‘(1) (a) A person who has knowledge that a sexual offence has been committed against a child must report such knowledge immediately to a police official.

(b) A person who fails to report such knowledge as contemplated in paragraph (a), is guilty of an offence and is liable on conviction to a fine or to imprisonment for a period not exceeding five years or to both a fine and such imprisonment’. (Criminal Law (Sexual Offenses and Related Matters) Act 32 0f 2007: 58)

This would mean that those dispensing contraceptives to teenage girls under the age of 16 would have to report sexual activity of girls with their partners as criminal, even if the intercourse was consensual. These contradictions between the Criminal Act and the Children’s Act posed a possible barrier to the dispensing of contraceptives on school grounds. Such obligations could make service providers reluctant to provide contraceptives altogether or this could make teenage girls avoid the use of the clinic in an attempt to protect themselves and their partners. Service providers were faced with the challenge of deciding whether to give contraceptives to teenagers and then report the statutory rape to the police as required by law, or rather to avoid this dilemma by not prescribing contraceptives to teenagers.

A recent High Court ruling held that aspects of sections 15 and 16 of the Sexual Offences Act infringed on the constitutional rights of adolescents outlined in the Children’s Act, by criminalizing consensual sexual activities between teenagers of a certain age group. The Constitutional Court ruled that these Sections were constitutionally inconsistent with the Children’s Act and therefore invalid. The matter was referred to Parliament (Khampepe, 2013), which has since ruled in favour of decriminalizing sexual acts between consenting teenagers (Government Gazette, 2015). These laws affected teenage girls aged younger than 16 years only, as these did not apply to persons older than 16 years of age. The recent court and parliamentary rulings removed the legal barriers to establishing a school-based contraceptive service for teenagers of all age groups. Service providers are now not required to report consensual sexual activity involving teenagers and will not be in violation of any laws when dispensing contraceptives to teenagers.

#### Policy provisions for an SBCC

Current Department of Health and basic Education policy makes good policy provision for the establishment of SBCCs, but all policies remain tentative in relation to school governing bodies. The ISHP (Department of Health, 2012) explicitly requires the provision of the full spectrum of SRH services for school-going adolescents, whether based at schools or at primary level health care services. The most recent DBE draft policy on HIV, STIs and TB (DBE, 2015), requires the provision of SRH services at schools, but retains the condition of such services being subject to the approval of individual school governing bodies. This draft policy therefore still endorses this contested condition, which is a potential barrier to the provision of any SRH at schools, including SBCCs.

### Views of teenage girls and parents of teenage girls towards an SBCC

Prevention of teenage pregnancy requires the understanding of factors that influence sexual behaviour and the use of contraceptives. We explored the remaining three components of the Social–Ecological Model (individual, interpersonal and community) to better understand enablers and barriers for an SBCC.

Teenage pregnancy was an important, prevalent issue of concern in this rural community as deemed by 97.5% of parent respondents. Teenage girls concurred, as they overwhelmingly expressed this in both FGDs.

Personal factors and beliefs are strong drivers for how the girls perceive sexuality and the use of contraceptives. The predominant opinions on teenage sexual activity amongst most of the 18 participants in the two FGDs were shaped by conventional morality. The majority of respondents felt that teenage sex was unacceptable and contrary to good moral upbringing and at times strong terms such as teenage sex being ‘slutty’ emerged.

As expressed by two respondents:

‘children of our age know they shouldn’t be having sex, so why do they have sex if they know what the consequences are?’ [F1]

‘if my mother raised me with the right morals and the right things then I wouldn’t go outside and have sex when I know it’s not my time and it’s not the place. Then I wouldn’t fall pregnant and it’s not necessary for my mother to put me on contraception’. [F12]

Their own moral judgment of themselves resulted in reluctance to access contraceptive services, as they felt ashamed and feared being judged by others.

For one of the respondents, religious beliefs strongly influenced decisions about contraceptives.

‘I can’t use contraception, because I am Catholic and contraception is forbidden’. [F5]

Despite these strongly expressed moral views; respondents acknowledged that pregnancies resulted from unplanned sexual events due to alcohol use, coercion and partners not wanting to use condoms in ‘the heat of the moment’. They cited a combination of peer pressure, pressure from their partners and prevailing social and economic pressures as drivers for sexual activity. These factors also influenced whether they would use contraceptives or not, in particular partner pressure was cited several times as a reason to refrain from using condoms in particular.

‘They tell you they don’t want to use condoms, and they tell you they will be safe, so then you say okay, I trust him’. [F11]

Interestingly, none of the girls openly admitted to being sexually active during the sessions, others admitted to knowing teenagers who engage in sexual activity and primarily assigned this practice to peer pressure. In addition, poverty was frequently cited as a contributing cause of teenage sexual activity and when asked to qualify what this meant, one of the respondents indicated that:

‘Like a lot of girls, they grow up without fathers, and the lack of love, and they tend to search for it in other places like trying to fall in love, and the guy usually misleads them, and then they try to have sex with the girl, or the girls try to fit in, in the crowd, go to parties and they drink and whatever, and when they’re drunk they do things that they’re not supposed to do and fall pregnant, or they are at parties and they’re not taking care of themselves, or they just drink anything that people offer them’. [F1]

It was clear in the discussions that, despite all the drivers for sexual activity, teenage girls did not wish to become pregnant. In most of their responses, participants intimated that those who are sexually active should use contraceptives, but at the same time cited a number of reasons why this practice does not occur. Some of the key factors that emerged from both focus groups included their moral and religious values; believing that they required parental consent for access to contraceptives; widespread concern about potential side effects and unfriendly service providers.

Whilst some girls felt that they required parental consent before starting on contraceptives, others asserted their autonomy and felt that it was their choice. One in particular stated that she would make the choice herself about when to start using contraceptives, regardless of what others might think. The majority of girls who spoke about this made reference to the influence of their mothers and intimated that maternal views strongly influenced their decisions about using contraceptives. These factors also influenced their likely use of an SBCC. Those whose parents were negative about contraceptives and sex, as well as those who did not openly speak about sex with their parents, were less likely to use the SBCC. Unsurprisingly their liberty of discussing sex with parents, in particular mothers, varied:

‘I don’t feel comfortable talking to my mom about things like that’. [F14]

‘My mommy and I have talked about everything. Like we have talked about sex and contraceptives, but it’s not something that she would tell me to use. She would leave the decision with me, but she trusts me in not doing something before my time’. [F13]

Real concerns about specific side effects, as well as the practicalities of using contraceptives, also emerged. The commonest ones cited were weight gain, impaired future fertility and intrauterine devices falling out.

When their perspectives on the appropriateness and influence of an SBCC were sought, girls had mixed reactions, in contrast to the parents who had strongly convergent views. Amongst the parent respondents, all felt that an SBCC could help to decrease teenage pregnancy, and an overwhelming 37 out of 40 agreed with the concept of an SBCC. However, similar to the perspectives of a number of the girls, 70% of parents also believed that the presence of an SBCC could promote promiscuity. Teenage respondents also held the view that the presence of an SBCC will send a message that ‘teenage sex was acceptable’. Both parents and teenage girls feared that having an SBCC, coupled with peer pressure, might encourage girls who are not yet sexually active to start engaging in sexual intercourse, as contraceptives would be easily available. In contrast, other parents and teenage girls saw an SBCC as a potential source of education that could result in fewer pregnancies and help teenagers to make better sexual choices.

The negative factors prevalent at primary health care clinics made the option of an SBCC more attractive. Lack of confidentiality from the clinic staff, unfriendly and judgmental staff attitudes, and clinic distance, coupled with long waiting times, resulted in some girls saying that they would not seek information at the local clinic and would feel more comfortable going to an SBCC. The girls almost unanimously agreed that an SBCC would be logistically more convenient and could decrease the rate of teenage pregnancy, as it would allow for easier access to contraceptives. This would also provide parents with peace-of-mind regarding the safety of their daughters in a gang-ridden community.

As stated by one of the parents:

‘I would feel reassured as my child would not have to walk that far and it would probably be safer (about an SBCC)’. [P7]

Potential lack of confidentiality by the SBCC providers would prevent teenage girls from using the service. However, if a school-based clinic offered general health services, including contraceptives, they would more readily make use of the service as no one would know why they visited the clinic. The girls also worried about what their friends, teachers and the community at large would think of them if seen using an SBCC. Parents feared that learners using the SBCC would be subjected to stigma and shame from fellow learners, leading to discontinued use of the service.

### Views from key informant interview

The school Principal confirmed some of the views expressed by parents and the teenage girls. He highlighted that teenage pregnancy was a problem at the school. Poor socio-economic factors as a key underlying driver for teenage pregnancy was re-iterated by the principal. Although he was in support of an SBCC, he worried that parents would not agree to the concept, for fear of it promoting promiscuity. Another concern was stigmatization of the learners by the community if it was known that the school hosted a contraceptive service.

## Discussion

This small study explored the views of school-going teenage girls and unrelated parents with teenage daughters in a low-income, rural community on teenage sexuality, teenage pregnancy and the desirability and feasibility of an SBCC, the likes of which exist in other parts of the world.

The many barriers faced by girls when trying to access local community-based clinics offset the potential negatives of an SBCC in this community. The teenage girls and parents with teenage daughters had surprisingly, strongly convergent views on a number of issues. They agreed on the desirability of a secure, easily accessible service at school where contraceptives could be obtained, but also held a view of potentially promoting promiscuity by allowing such a service. Another issue was that confidentiality would be difficult to maintain and girls making use the service would be stigmatized. A recent study conducted in 33 South African public schools looking at condom distribution at schools found that teenagers expressed the same concerns regarding confidentiality, privacy, stigmatization and increased promiscuity (de Bruin and Panday-Soobrayan, 2017). The promotion of promiscuity through the presence of an SBCC is not a uniquely held view, as a US-based study explored this issue to dispel US parent fears. Results showed no association between the establishment of an SBCC and increased promiscuity (Foster, 1999). Parents and girls thought that an SBCC could work, provided that the school health service offered a range of services, including sexual health services.

In keeping with studies debunking widely held views amongst many groups in South African society that teenagers wilfully fall pregnant to obtain a child support grant (Kubheka, 2013); the girls in this study were uniformly clear that neither they, their friends, nor the teenage boys that they knew, wished to become parents, and that in their view teenage pregnancy was usually unplanned and undesirable. Like many teenagers, the participants in this study were conscious of how others would perceive them, and were particularly concerned about being viewed as ‘sluts’. Nonetheless, peer pressure to engage in sexual activity remains an issue.

The preference of participants in this study was for a general school-based service, that included SRH components, whilst strongly upholding confidentiality. Parents felt it was safer than going to clinics in the community, and girls felt that it would address many of the barriers faced when accessing clinics, which includes service provider judgment and abuse towards them. However, despite the strongly supportive legislative framework for school-based SRH services, the provision of a permanent on-site SBCC that would be available at every school on a daily basis, and not the once or twice a year visits by school health teams as is currently the practice (Shung -King, 2013), is an unlikely option in the immediate future. There is, however, an indisputable need for an acceptable, easily accessible, SRH service to teenage schoolgoing girls.

While the study primarily explored the perceptions about an SBCC, the use of the ecological framework in constructing the study questions inadvertently resulted in teenagers and parents highlighting multiple factors that posed barriers to accessing SBCC and SRH services more broadly. In keeping with available literature on this subject in South Africa and the long-standing discussions and debates that the authors have been privy to, we wish to highlight some key suggestions and recommendations for the establishment of SBCC and SRH services more broadly.

The location, nature and functioning of such a service must still be resolved. The ISHP clearly outlines the spectrum of services that should be available, but whether this should be provided by the currently roving school health teams, or by local clinics with a strong link to local schools, or possibly be located in a community site that is neither at a school nor a clinic, are options under consideration (Mathews *et al*., 2015; Mason-Jones *et al*., 2016). Success in establishing an SRH service that will meet the needs of schoolgoing teenagers will require collaboration between the Department of Health and the DBE and a well-coordinated response between the ISHP, the school, local clinics and relevant non-governmental organizations who work in this area (Shung-King, 2013). A possible barrier to this success lies in the discretion of school’s governing bodies as to whether SRH services could be provided at schools or not. This decision is influenced by who serves on the governing body and what their worldviews are (Gray and Vadwa, 2014).

The study highlights the importance of gaining multiple perspectives on issues as complex as teenage sexuality and pregnancy, and very importantly the views of young people themselves. This is a crucial message to policy makers and health service planners who are the custodians of the design and delivery of services. The results suggest that multiple factors operating at different levels of a Social–Ecological Model influence the presence, design, access and use of a service such as an SBCC. For successful implementation of the SBCC, all these factors need to be attended to at the same time.

The findings further point to the need for greater education and awareness to promote healthier attitudes and beliefs towards teenage sexuality and the use of contraceptives, whilst at the same time highlighting the health and social detriments of teenage pregnancies for the teenagers themselves, their babies and their families. SRH is already taught in South African schools. Participants in this study highlighted that an SBCC would further supplement this education and serve as a catalyst for change in behaviour. It requires a multipronged approach that will focus on the teenagers themselves, their parents, caregivers, the school community and the community at large, as well as health service providers. The training of service providers in particular is crucial in ensuring a more positive and non-judgmental approach when assisting teenage girls with contraceptives.

The ethical dilemma faced by service providers and stakeholders due to the contradiction between the Sexual Offenses Act and the Children’s Act has been alleviated by the Parliament’s ruling that consensual intercourse between teenagers of certain age groups will not be criminalized. However, the ISHP is still in contradiction to the Children’s Acts which allows children to consent to contraceptives without their parent’s knowledge from the age of 12 (14 years in the ISHP). This raises a potential area of conflict between services providers and stakeholders. Although the ISHP could facilitate the establishment of an SBCC, many implementation challenges still exist, including the dependency on permission of school governing bodies, and capacity of health services to support an SBCC (Shung-King, 2013). While attempts have been made to ensure that these factors are addressed in the new policy, they still remain a threat.

This was a small study with a number of limitations: We opted to only focus on girls, and had boys been interviewed, we may have elicited different results to those obtained from the girls. The study by De Bruin and Panday-Soobrayan published in 2017, interestingly revealed a more conservative set of views by boys compared to girls. It is not possible to say whether the girls felt pressured to answer in a particular way after being selected by their Life Orientation teacher to participate in the focus group and selection bias, is also difficult to rule out. However, the variety of responses received and the consistency of responses across both focus groups, suggest that the girls were expressing independent opinions. Proof that the Life Orientation teacher adhered to the criteria of selecting girls of varying backgrounds and academic achievement for the FGDs was not explored. Parental factors that could have influenced responses for and against an SBCC (such as number of children) were not explored. We suggest that a more detailed research study into perspectives of a wider range of teenage girls and boys about an SBCC should be conducted. This should also include views from school governing body members, educators, more male parents and primary health care service providers, which may provide a more comprehensive set of views on the acceptability of an SBCC, as well as ideas on how to implement such a service in a sustainable way.

## Conclusion

This study confirmed that teenage girls in this South African setting face many pressures in terms of perceptions about sex and sexual activity. Barriers to contraceptive services whether school-based or clinic-based persist and impacts on girls’ willingness and ability, to access contraceptives. Whilst parents with teenage daughters and the focused group participants were mostly in favour of an SBCC, as well as legal and policy statutes of being in favour such a service: provision of an SBCC on a permanent basis, is not imminently likely or possible due to the many resource and logistical constraints. Alternative models must be explored, which may include a school-linked service with integral support from primary health care and appropriate non-governmental services.

## References

[ref1] Abdool Karim S, Baxter C, Frohlich J Abdool Karim Q (2014) The need for multipurpose prevention technologies in Sub-Saharan Africa. British Journal of Obstetrics and Gynaecology 121, 27–34. 25335838 10.1111/1471-0528.12842 PMC4206830

[ref2] Alexander M, Farmer M, Hotra D, Johnson T, Papa P, Rosenthal B Santelli J (1992) Bringing parents into school clinics: parents attitudes towards school clinics and contraception. Journal of Adolescent Health 13, 269–274. 1610841 10.1016/1054-139x(92)90158-8

[ref3] Attride-Stirling J (2001) Thematic networks: an analytic tool for qualitative research. Qualitative Research 1, 385–405, DOI: 10.1177/146879410100100307

[ref5] Brindis CD, Keeton V Soleimanpour S (2012) School-based health centers in an era of health care reform: building on history. Current Problems in Paediatric Adolescent Health Care 42, 132–156. 10.1016/j.cppeds.2012.03.002 PMC3770486 22677513

[ref6] Brodmann S Douglas SM (2016) Spheres of influence: the social ecology of racial and class inequality. Sociological Inquiry 86, 98–99.

[ref7] Chaudhuri RN, Mukhopadhyay P Paul B (2012) Hospital based perinatal outcomes and complications of teenage pregnancy in India. Journal of Health Population and Nutrition 28, 484–500. 10.3329/jhpn.v28i5.6158 PMC2963772 20941901

[ref8] Christofides NJ, Dunkle KL, Jewkes RK, McCarty F, Nduna M, Shai NJ Sterk C (2014) Risk factors for unplanned and unwanted teenage pregnancies over two years of follow up among a cohort of young South African women. Global Health Action. Retrieved 23 December 2017 from www.globalhealthaction.net/index.php/gha/article/view/23719 10.3402/gha.v7.23719 PMC4141943 25150027

[ref10] Crisp C, Koech J, De Koker P, Manson-jones AJ, Mathews C Momberg M (2012) School-based health clinics for adolescent sexual, reproductive and mental health. BMC Systematic Reviews 1, 49. 10.1186/2046-4053-1-49 PMC3621403 23098138

[ref11] Daniels JA, Doyle LS Zimmer-Gembeck MJ (2001) Contraceptive dispensing and selection in school-based health centers. Journal of Adolescent Health 29, 177–185. 11524216 10.1016/s1054-139x(01)00220-8

[ref12] Department of Basic Education (2015) Draft Department of Basic Education national policy on HIV, STIs and TB (pp. Government Gazette 395).

[ref13] de Bruin WE Panday-Soobrayan S (2017) Learners’ perspectives on the provision of condoms in South African public schools’. AIDS Care 12, 1529–1532. 10.1080/09540121.2017.1327647 28509570

[ref14] Department of Health and Department of Basic Education (2012) Integrated School Health Policy. Retrieved 14 December 2017 from https://cs.mg.co.za/content/documents/2017/06/14

[ref15] Departments of Health and Basic Education (2012) Integrated school health programme resource manual for School health nurse. Retrieved 15 December 2017 from www.section27.org.za/wpcontent/uploads/2013/01/IntSchoolHealthProg.pdf

[ref16] Earp JL Golden SD (2012) Social ecological approaches to individuals and their contexts. Health Education & Behavior 39, 364–372. 22267868 10.1177/1090198111418634

[ref17] Ethier KA, Chung EQ, DeRosa CJ, Dittus PJ, Kerndt PR Martinez E (2011) School-based health centre access, reproductive health care, and contraceptive use among sexually experienced high school students. Journal of Adolescent Health 48, 562–565. 21575814 10.1016/j.jadohealth.2011.01.018

[ref37] Foster N (1999) School Based Health Centers. Advoctes for Youth. Retrieved 24 June 2017 from www.advocatesforyouth.org/publications/publications-a-z/448-school-based-health-centers

[ref18] Ganchimeg T, Ota E, Morisaki N, Laopaiboon M, Lumbiganon P, Zhang J, Yamdamsuren B, Temmerman M, Say L, Tunc O, Vogel JP, Souza JP Mori R, on behalf of the WHO Multicountry Survey on Maternal Newborn Health Research Network (2014) Pregnancy and childbirth outcomes among adolescent mothers: a World Health Organization multicountry study. British Journal of Obstetrics and Gynaecology 121, 40–48. 10.1111/1471-0528.12630 24641534

[ref19] Government Gazette (2015) Criminal law (sexual offences and related matter) amendment act, vol. 601, No. 38977. Retrieved 20 December 2017 from www.justice.gov.za/legislation/

[ref20] Government Gazette, (2005) Children’s Act, Act No. 38. Retrieved 20 December 2017 from www.justice.gov.za/legislation/acts/2005-038%20childrensact.pdf

[ref38] Gray A and Vawda Y (2017) Health Policy and Legislation. In Padarath A, Barron P, editors. *South African Health review* Retrieved 24 November 2017 from www.hst.org.za/publications/south-african-health-review-2017

[ref21] Han J Bennish ML (2009) Condom access in South African Schools: law, policy, and practice. *PLoS Medicine* **6**, e1000006. 10.1371/journal.pmed.1000006 PMC2628401 19166265

[ref22] Khampepe J, Mogoeng CJ, Bosielo AJ, Froneman J, Jafta J, Mhlantla AJ, Nkabinde J, Skweyiya J Zondo J, concurring (2013) Constitutional Court of South Africa Case CCT 12/13 ZACC 35.

[ref23] Kubheka ZL (2013) The relationship between child support grant and teenage pregnancy, Masters dissertation. KwaZulu Natal, South Africa: University of Zululand.

[ref24] Lee LRT (2011) The role of school nurses in delivering accessible health services for primary and secondary school students in Hong Kong. Journal of Clinical Nursing 20, 2968–2977. 21777311 10.1111/j.1365-2702.2011.03782.x

[ref25] London L, Morojele N Peer N (2013) Factors associated with contraceptive use in a rural area in Western Cape province. South African Medical Journal 103, 406–412. 23725962 10.7196/samj.6201

[ref27] Mason-Jones AJ, Sinclair D, Mathews C, Kagee A, Hillman A Lombard C (2016) School-based interventions for preventing HIV, sexually transmitted infections, and pregnancy in adolescents. *Cochrane Database of Systematic Reviews*, Issue 11. Art. No.: CD006417. DOI: 10.1002/14651858.CD006417.pub3 PMC5461872 27824221

[ref28] Mathews C, Eggers SM, de Vries PJ, Mason-Jones AJ, Townsend L, Aaro LE De Vries H. (2015) Reaching the hard to reach: longitudinal investigation of adolescents’ attendance at an after-school sexual and reproductive health programme in Western Cape, South Africa. BMC Public Health 15, 608. 26141155 10.1186/s12889-015-1963-3 PMC4490658

[ref39] South African Marketing Research Association (2015) Guideline for Research with Children in South Africa. Retrieved 15 April 2017 from www.samra.co.za/saethicschidren/

[ref29] Statistics South Africa (2016) South Africa Demographic and Health survey. 2016: Data Quality report. Retrieved 17 December 2017 from https://www.statssa.gov.za/publication/report%252003-00-09/report

[ref30] Shung-King M (2013) From ‘stepchild of primary healthcare’ to priority programme: lessons for the implementation of the National Integrated School Health Policy in South Africa. South African Medical Journal 103, 895–898. 24300623 10.7196/samj.7550

[ref31] Strode A, Slack C Essack Z (2011) Child consent in South African law: implications for researchers, service providers and policy-makers. South African Medical Journal 101, 604–606. 21920172

[ref32] Western Cape Government (2012) Regional development profile Overberg district. Retrieved 15 December 2017 from https://www.westerncape.gov.za/assets/departments/treasury/dc03_overberg_district_regional_profile_2012_nov_2012.pdf

[ref33] The World Bank: Adolescent fertility rate (births per 1000 women ages 15–19) (2014) 14 December 2017 from http://data.worldbank.org/indicator/SP.ADO.TFRT

[ref34] Willan S (2013) A review teenage pregnancy in South Africa of partners in sexual health. Retrieved 18 December 2017 from http://lifeline.ume.co.za/uploads/files/news%20folder/TeenagePregnancy2013.pdf

[ref35] World Health Organization (2014) Adolescent pregnancy. Retrieved 18 December 2017 from https://www.who.int/mediacentre/factsheets/fs364/en/

[ref36] Zuch M, Mason-Jones AJ, Mathews C Henley L (2012) Changes to the law on consent in South Africa: implications for school-based adolescent sexual and reproductive health research. *BMC International Health and Human Rights* 12, 3. 10.1186/1472-698X-12-3 PMC3353180 22490444

